# Galectin-1 in Obesity and Type 2 Diabetes

**DOI:** 10.3390/metabo12100930

**Published:** 2022-09-30

**Authors:** Emanuel Fryk, Vagner R. R. Silva, Per-Anders Jansson

**Affiliations:** Department of Molecular and Clinical Medicine, Institute of Medicine, Sahlgrenska Academy, University of Gothenburg, 413 45 Gothenburg, Sweden

**Keywords:** galectin-1, obesity, insulin resistance, type 2 diabetes

## Abstract

Galectin-1 is a carbohydrate-binding protein expressed in many tissues. In recent years, increasing evidence has emerged for the role of galectin-1 in obesity, insulin resistance and type 2 diabetes. Galectin-1 has been highly conserved through evolution and is involved in key cellular functions such as tissue maturation and homeostasis. It has been shown that galectin-1 increases in obesity, both in the circulation and in the adipose tissue of human and animal models. Several proteomic studies have independently identified an increased galectin-1 expression in the adipose tissue in obesity and in insulin resistance. Large population-based cohorts have demonstrated associations for circulating galectin-1 and markers of insulin resistance and incident type 2 diabetes. Furthermore, galectin-1 is associated with key metabolic pathways including glucose and lipid metabolism, as well as insulin signalling and inflammation. Intervention studies in animal models alter animal weight and metabolic profile. Several studies have also linked galectin-1 to the progression of complications in diabetes, including kidney disease and retinopathy. Here, we review the current knowledge on the clinical potential of galectin-1 in obesity and type 2 diabetes.

## 1. Introduction

Galectin-1 is a protein with carbohydrate-binding capabilities, expressed in several cell types in the body. Galectins are a family of proteins, numbered 1–15 in order of discovery, that are highly conserved between mammals [1,2]. A common trait of the galectins is the ability to bind galactose, but galectins also bind several other sugar molecules in the body, enabling interaction with glycoproteins and glycolipids. One carbohydrate structure known to interact with galectins is the disaccharide N-acetyl-lactosamine, found on many different glycoproteins and glycolipids in the human body [1,3].

Galectin-1 is involved in key cellular functions related to tissue maturation, homeostasis and remodelling through regulation of cell migration, proliferation, angiogenesis, apoptosis, inflammation, among others [1]. The interest in galectins in human disease has increased over the recent years, and several comprehensive reviews have previously provided overviews on the effects of galectins in various conditions [1,2,4,5,6,7,8,9,10,11,12]. Recently, several galectins, including galectin-1, have also been identified as important for adipose tissue homeostasis, both as regulators of adipose tissue function, and in metabolic disease [7,13,14,15,16,17,18,19,20,21]. Galectin-1 is expressed in several human tissues, with the highest expression in subcutaneous adipose tissue, followed by blood vessels and female reproductive organs [15]. Galectin-1 gene expression has been shown to be increased in adipocytes from obese mice, and in isolated adipocytes from type 2 diabetes patients (T2D) [15,22]. In addition, several independent research groups have reported a link between galectin-1 expression in the adipose tissue and metabolic outcomes in different animal models [23,24,25].

The adipose tissue is an important organ in normal physiology. Besides storing excess energy in the form of triglycerides, adipose tissue also has other significant functions. Metabolically, adipose tissue releases fatty acids to fuel other tissues through the process of lipolysis and secretes several proteins into the circulation [26]. These proteins, often referred to as adipokines, can present both local and systemic effects. The study of adipokines is central for providing new insights concerning the underlying pathophysiology of obesity and related metabolic aberrations. High body mass index (BMI) is strongly correlated with an increased burden of disease, and premature death [27]. Similarly, an increased adiposity is associated with an increased risk of several adverse outcomes including cardiovascular disease, T2D and cancer, among others [28,29,30,31,32]. Recently, obesity was also related to complications after a primary infection with the COVID-19, with an increased risk of morbidity and mortality [33]. It is well known that the number of individuals with obesity is globally increasing, with an estimated 107.7 million children and 603.7 million adults meeting the definition of obesity (BMI ≥ 30 kg/m^2^) [27]. Despite continuous advances in science, obesity and its consequences are still a public health problem.

T2D increases globally, as a consequence of the obesity pandemic [34,35,36]. It is predicted that 642 million people will have diabetes in 2040, and 90% will be T2D cases [37]. A large proportion of individuals with T2D do not know that they have the disease [38]. Early detection, improved treatment, and an improved understanding of the disease is important to minimize adverse events, reduce healthcare costs, and reduce the debilitating effects. T2D doubles the risk of several cardiovascular diseases, including myocardial infarction and cerebral stroke [39]. Recent years have seen a change in the clinical approach to T2D, novel approaches to the delivery of drugs have appeared, and the use of GLP-1 analogues and SGLT2-inhibitors have demonstrated that new treatment alternatives can still influence the overall care of patients with T2D and that the assessment of new treatment targets is still important [40,41]. In line with this, animal studies have assessed the feasibility of modulating galectin-1 as a treatment alternative in obesity and complications in T2D [25,42,43]. T2D is also one of the biggest risk factors for chronic kidney disease, and several vascular complications including retinopathy and lower leg amputation [44,45,46]. In recent years, several studies have examined the potential use of circulating galectin-1 as a potential biomarker in T2D and predictor of adverse events and explored its possible role in modulating kidney disease [43,47,48]. While galectin-1 does not currently have a place in clinical practice, it is being studied in these areas. Several pharmacological agents targeting galectin-1 are currently in development, and galectin-3 inhibitors with similar molecular structure are already in phase III clinical trials [4,6].

T2D is caused by a combination of genetic and environmental factors, where sedentary lifestyle and a high intake of saturated fats and processed sugars are major contributors [49,50,51]. T2D is often preceded by obesity, in itself a major risk factor for T2D, and these two conditions share the same risk factors [52,53,54]. It is hypothesized that a chronic positive energy balance eventually will result not only in obesity, but also insulin resistance and T2D, particularly in genetically predisposed individuals [55]. Mechanistically, a sustained positive energy balance could explain the pathophysiological link between obesity and T2D. Excess energy intake combined with a saturated capacity to store additional energy in natural fat depots eventually results in ectopic fat deposition [56,57,58]. When traditional adipose tissue depots are unable to respond to an increased need for triglyceride storage, ectopic fat deposition results in increased triglyceride content in organs such as the liver, pancreas, and muscle. These organs are all central in regulating energy balance and maintaining a glucose-insulin homeostasis [59,60]. Alongside ectopic fat deposition, an impaired response in the glucose lowering effect of insulin is often seen, referred to as systemic insulin resistance. The progression from obesity to insulin resistance and eventually T2D is an established model of disease in the development of T2D and the adipose tissue is believed to have a central role in this progression [61,62,63]. Studies in humans have been demonstrating an association between galectin-1 and lifestyle, genetics as well as insulin resistance in recent years, underlining a potential role of galectin-1 in these processes [15,47,64,65]. Here, we review pre-clinical and clinical knowledge surrounding galectin-1 in obesity, its relation to mechanisms in insulin resistance with emphasis on clinical studies in T2D and its complications. Original papers on galectin-1 and obesity, insulin resistance or type 2 diabetes are summarized in Table 1.

## 2. Galectin-1 in the Adipose Tissue and in Obesity

Over the past decades since the discovery of the hormone leptin, the adipose tissue is considered to be an endocrine organ that can secrete and release hormones and adipokines important for modulation of satiety, inflammation, immune cells and lipid metabolism [66,67,68]. Recently, galectin-1 and its important role in adipose tissue homeostasis as a proposed adipokine has been gaining support in the literature. Several studies have identified that galectin-1 subcutaneous adipose tissue expression is altered during changes in body weight. Galectin-1 gene expression decreased in obese participants during weight loss by very low-calorie diet (VLCD). The same study also reported that *LGALS1* increased during weight gain through high calorie diet [15]. Similar results were also reported after a dietary intervention, five weeks (500 kcal per day) or twelve weeks (1250 kcal per day), in healthy and overweight/obese subjects where a clustering correlation analysis associated high galectin-1 expression with an increased risk of weight regain [65].

**Figure 1:**
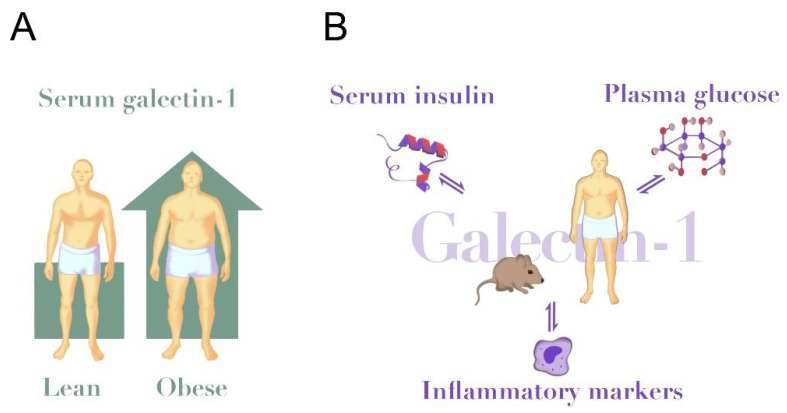
Galectin-1 in obesity and insulin resistance. (**A**) Circulating galectin-1 levels are elevated in human obesity (~25% higher in obese vs. lean [64]) and in HFD-fed mice (~50% higher in HFD vs. chow [22]). (**B**) Circulating galectin-1 is positively associated with serum insulin levels in humans and modulated insulin secretion in mice [64,80]. Further, galectin-1 inversely associates with plasma glucose [64], but galectin-1 reduced glucose uptake in human adipose cells in vitro [15]. Insulin and glucose confer direct stimulatory effects on galectin-1 secretion to cell media from several cell types [71,77,80,94,95,96]. Galectin-1 is positively associated with circulating levels of IL-6, TNF-α and CRP [47,64]. Likewise, triggers of inflammatory pathways up-regulate galectin-1 in several immune cells [97].

Circulating galectin-1 was also associated with obesity in several studies in both humans and in obese animal models [22,64,69] (Figure 1A). In a cross-sectional population-based cohort study with 989 participants aged 50–65 years, galectin-1 increased in both female and male subjects when participants were stratified using BMI into lean, overweight, and obese. Galectin-1 also correlated with both BMI and plasma triglyceride concentration, and inversely with plasma high-density lipoprotein cholesterol (HDL) [64]. Similarly, serum galectin-1 levels were higher in obese children compared to lean children [69]. In correlation analyses, galectin-1 appeared to be positively correlated with fat mass. The authors concluded that galectin-1 levels were proportional to the fat mass in children with obesity but not in the lean group [69]. A clinical study also identified that galectin-1 is secreted to the extracellular space in human subcutaneous adipose tissue, noting that the local interstitial galectin-1 concentrations may be 10–20 times higher than the circulating level of galectin-1 [15].

In line with observations reported in clinical studies, several studies in animal models have also identified increased expression of galectin-1 in obesogenic conditions. Studies including high-fat diet (HFD) or *ob/ob* mice (leptin knockout mice, a classic model to study obesity in animal models) have all presented increased galectin-1 in the adipose tissue [22,70]. Furthermore, galectin-1 knockout as well as galectin-1 inhibition has been demonstrated to prevent weight gain in animals fed with HFD [22,23,24,70]. Taken together, these studies suggest that galectin-1 is not only associated with obesity, but also has a functional role in adipose tissue physiology and regulation of fat mass homeostasis. 

**Table 1:** Original papers on galectin-1 in association with obesity, insulin resistance and type 2 diabetes identified through a structured search in PubMed in August 2022 using the following terms: galectin-1 and obesity, galectin-1 and insulin resistance, galectin-1 and type 2 diabetes, totalling 23 publications.

Ref.	Studied Model	Key Methods	Key Results
Liu X et al. 2009 [71]	17 T2D patients and 15 lean controls. *In vitro* analyses.	(L6) cells. Proteomic analysis and ELISA in plasma.	Galectin-1 protein expression increased in samples from T2D patients. L6 cells treated with glucose up-regulated galectin-1 protein expression.
Ahmed M et al. 2010 [72]	10 moderately obese, but otherwise healthy, subjects.	Treatment with rosiglitazone for 14 days; proteomic analysis of changes in abdominal subcutaneous adipose tissue.	Rosiglitazone increased galectin-1 protein expression.
Pini M et al. 2013 [73]	Control and high-fat DIO mice and IL-6^−/^^−^ mice; LPS treatment.	LPS was administered i.p. Blood was obtained from the retroorbital plexus in separate groups of mice.	Plasma galectin-1 was suppressed by LPS treatment and obesity and IL-6^−/−^ knockout modulated the response.
Mukherjee R et al. 2015 [70]	Mouse 3T3-L1 and HIB1B preadipocytes; primary white adipocytes isolation; TDG treatment; gal-1 silencing (siRNA). Control and HFD rats.	3T3-L1 and HIB1B preadipocytes were cultured and differentiated; primary white adipocytes were isolated from epididymal WAT depots of rats and cultured; both immortalized and primary adipocytes were treated with TDG; i.p. of TDG once per week for 5 weeks in rats.	Galectin-1 silencing attenuated adipogenesis and lipogenesis in both 3T3-L1 and HIB1B adipocytes. Treatment with TDG, to cultured adipocytes in vitro reduced fat accumulation. IP injection of TDG resulted in dramatic inhibition of HFD-induced body weight, reduced adipogenesis and lipogenesis, increased expression of proteins associated with thermogenesis and energy expenditure.
Mukherjee R et al. 2015 [25]	*In vitro* analyses; treatment with LBA; NC and HFD rats.	3T3-L1 and HIB1B preadipocytes were cultured and differentiated; Rats were divided into 4 groups: NC, HFD, HFD-fed rats treated with LBA by LBA-OR, and HFD-fed rats treated with LBA by LBA-IP.	LBA treatment reduced lipogenic capacity of both 3T3-L1 and HIB1B adipocytes through down-regulation of major adipogenic transcription factors at both mRNA and protein levels. LBA-OR and LBA-IP reduced body weight gain, suppressed lipogenic transcription factors and attenuated lipogenesis and fat accumulation.
Parray, HA; Yun, JW. 2015 [74]	Control and HFD Sprague Dawley rats; inhibition of galectin-1 by TDG treatment.	Treatment with 5 mg/kg of TDG for 5 weeks (once per week). Proteomic analyses in the WAT.	CA3, VDAC1, PEBP1, ANXA2 and LDHA protein levels between WAT from control and TDG-treated groups. Increased expression of thermogenic proteins, reduced expression of lipogenic proteins.
Mukherjee R et al. 2016 [24]	3T3-L1 and HIB1B cells; NC and HFD Sprague Dawley rats.; inhibition of galectin-1 by lactulose treatment.	3T3-L1 cells and HIB1B preadipocytes, respectively were cultured and differentiated in the presence of LT; NC, HFD-fed HC, HFD-fed rats treated with LT by oral administration (LT-OR), and HFD-fed rats treated with LT by i.p injection (LT-IP).	LT treatment reduced adipogenesis and fat accumulation *in vitro* by down-regulation of adipogenic transcription factors such as C/EBPα and PPARγ. *In vivo* treatment of LT blocked HFD-induced body weight gain and food efficiency and improved metabolic variables in the plasma. Reduced adipogenic (C/EBPα and PPARγ) and increased energy expenditure and lipolysis marker proteins (ATP5B, COXIV, HSL, and CPT1) in adipose tissue.
Fryk E et al. 2016 [15]	7 lean/overweight T2D patients and 8 lean controls.	Proteomics on the interstitial fluid of SAT microdialysis. Adipose tissue and isolated adipocytes from SAT biopsies.	Galectin-1 protein in subcutaneous dialysates and mRNA levels in adipocytes were elevated in T2D patients compared with healthy controls.
Parray, HA; Yun, JW. 2017 [75]	HFD CON male Sprague Dawley rats; 3T3-L1 and HIB1B cells; inhibition of galectin-1 by TDG inhibitor treatment.	HFD rats were divided into 2 groups: 5 mg/kg of TDG once per week for 5 weeks i.p. or controls; 3T3-L1 cells and HIB1B preadipocytes, respectively were cultured and differentiated.	TDG treatment reduced weight gain and fat mass, activated thermogenic markers in WAT and BAT, reduced protein levels of LC3-II and increased protein levels of P62. Combined inhibition of galectin-1 and ATG5 by TDG treatment protected against both HFD-induced adipogenesis and lipogenesis and blocked C/EBPα, PPARγ and FASN.
Roumans NJT et al. 2017 [65]	61 overweight and obese subjects underwent a DI.	Participants were randomly assigned to a VLCD (rapid weight loss) or an LCD (slow weight loss) group. SAT biopsy, transcriptomics and proteomics.	The galectin-1 gene (*LGALS1*) expression in SAT during DI correlated with risk of weight regain.
Acar S et al. 2017 [69]	45 obese and 35 lean children.	Obese children (mean age: 12.1 ± 3.1 years) and normal-weight children (mean age: 11.8 ± 2.2 years).	Serum galectin-1 levels were higher in obese children (12.4 ± 2.3 ng/mL) than those in normal-weight children (10.1 ± 1.6 ng/mL, mean ± SD). Galectin-1 correlated negatively with fasting glucose and positively with fat mass and waist circumference.
Williams, SP et al. 2017 [76]	*In vitro* analyses of human dermal microvascular neonatal LECs.	Migration of LECs. Genome-wide siRNA screen analyses.	*LGALS1* promoted lymphatic vascular growth in vitro and in vivo contributing to maintenance of the lymphatic endothelial phenotype. Signalling network for lymphangiogenesis and lymphatic remodelling presented.
Al-Obaidi N et al. 2018 [77]	MCT cells and HEK293 cells; galectin-1 inhibitor (OTX-008); Wild-type, Akita, db/db mice, galectin-1^−/−^ mice.	Cells were treated with increasing concentrations of glucose (HG) or high insulin (HI) for 72 h. Cells were pre-treated with OTX-008 or Akt inhibitor before exposure to HG or HG + HI. Mice were divided into 3 groups of 4 mice/group.	Tubular renal cells exposed to HG increased phosphorylation of Akt and galectin-1. OTX-008 decreased p-Akt/AP4 and protein-promoter activity of galectin-1 and fibronectin. Kidney of gal-1^−/−^ mice expressed very low levels of fibronectin protein. Galectin-1 may be a fibrosis protein.
Waltl I et al. 2018 [78]	17 with T2D and DME and 19 controls.	T2D and DME subjects were each treated with a single intravitreal injection of aflibercept monthly for 3 consecutive months.	Plasma galectin-1 levels increased to 27.0, 24.0 and 36.0 ng/mL at 7 days, 4 weeks and 8 weeks, respectively. Galectin-1 blocking antibodies may be useful in antiangiogenic therapy.
Fryk E et al. 2019 [64]	989 subjects aged 50–64 years; cross sectional study.	Analysis of serum levels of galectin-1 and circulating biomarkers.	Galectin-1 was independently and inversely associated with type 2 diabetes and glucose and positively associated with age, BMI, metabolic and inflammatory markers.
Tsai YT et al. 2019 [79]	*In vitro* analyses in cancer cells and *in vivo* analysis in mice models.	Human lung cancer cell line CL1-5 and tumour inoculation in mice. Galectin-1-targeting DNA aptamer (AP-74 M-545).	Immunohistochemistry revealed increased CD4^+^ and CD8^+^ T cells in AP-74 M-545-treated tumour tissues. AP-74 M-545 suppresses T cell apoptosis by blocking the binding of Galectin-1 to CD45, the main receptor and apoptosis mediator of galectin-1 on T cells.
Sundblad V et al. 2021 [80]	C57BL/6 *Lgals1^−/−^* mice; hand-picked islets from 5- to 6-month-old mice.	Male and female *Lgals1^−/−^* mice; metabolic phenotyping including food intake and glucose-stimulated insulin secretion (GSIS) in islets *in vitro*.	Lgals1^−/−^ female mice exhibited higher body weight, increased food intake, altered glucose tolerance and higher basal glucose levels. Further, GSIS was impaired while pancreatic insulin content was enhanced. Recombinant galectin-1 enhanced GSIS *in* *Lgals1^−/−^* islets.
Jovanovic MM et al. 2021 [81]	72 patients with metabolic syndrome and UC; observational cross-sectional study.	Concentrations of pro- and anti-inflammatory cytokines in serum and faeces samples were measured.	The enhanced inflammation in UC-patients in the terminal phase of the metabolic syndrome may be due to a decreased immunomodulatory influence of galectin-1.
Wu Z et al. 2021 [82]	C57BL/6 mice, adipose specific CD146 KO.	CD146 ablation in preadipocytes and mature adipocytes; BAT cells; human adipose tissue samples.	Adipose CD146 KO inhibits HFD-induced obesity. Galectin-1 inhibits UCP1 expression in BAT via CD146 by enhancing AKT and FoxO1 phosphorylation.
Baek et al. 2021 [23]	3T3-L1 and HEK293 cells; ND and HFD obese *Lgals1*^−/−^ C57BL/6 mice.	3T3-L1 cells were maintained and differentiated; ND and HFD *Lgals1*^−/−^ C57BL/6 mice were fed for 10-weeks.	Galectin-1 mRNA increased in muscle and adipose tissues of HFD mice. Galectin-1 increased during adipocyte differentiation and galectin-1 silencing inhibited PPARγ, C/EBPα, FABP4, and FASN. Lgals1^−/−^ mice fed HFD reduced body weight gain.
Drake I et al. 2022 [47]	Population-based cohorts; longitudinal studies.	4022 participants in the Malmö Diet and Cancer Study–Cardiovascular Cohort (MDCS-CC) enrolled between 1991–1994; All New Diabetics in Scania (ANDIS) enrolled 2007–2016 (n = 9367).	Serum galectin-1 at baseline predicts incident T2D at follow-up 18 years later. Galectin-1 is strongly associated with lower eGFR. MR analyses showed no causal effect of galectin-1 on CKD or T2D, but T2D patients from ANDIS belonging to SIRD subgroup showed genetically elevated galectin-1 in association with higher eGFR.
Luftmann BB et al. 2022 [83]	31 patients with COPD.	Presence of Tregs in BALF and peripheral blood; clinical phenotyping.	Serum galectin-1/TP was positively associated with % of Tregs in BALF.
Lluch A et al. 2022 [84]	Control and DIO mice.	Control and DIO mice were treated by oral gavage with LY2584702 tosylate (LY), S6K1 inhibitor, for 3 months.	LY reduced gene expression of LGALS1 in the liver and in subcutaneous adipose tissue in obese mice. Modulation of S6K1 may be a target for treatment of obesity, dyslipidaemia and liver steatosis in humans.

ANXA2, annexin A2; BALF, broncho alveolar lavage fluid; BAT, brown adiopose tissue; CA3, Carbonic anhydrase 3; CKD, chronic kidney disease; COPD, chronic obstructive pulmonary disease; DI, diet intervention; DIO, diet induced obesity; DME, diabetic macular edema HEK293, human embryonic kidney 293; HFD, high-fat diet; i.p., intraperitoneally; KO, knock out; LBA, lactobionic acid; LBA-IP, lactobionic acid – intraperitoneal injection; LBA-OR, lactobionic acid – oral administration; LCD, low calorie diet; LDHA, lactate dehydrogenase A chain; LECs, lymphatic endothelial cells; LT, lactulose; MCT, murine proximal tubular; NC, Control; PEBP1, phosphatidylethanolamine-binding protein 1; SAT, subcutaneous adipose tissue; T2D, type 2 diabetes; TDG, thiodigalactoside; Tregs, regulatory T cells; UC, ulcerative colitis; VDAC1, Voltage-dependent anion channel 1; VLCD, very low calorie diet; WAT, white adipose tissue; L6, skeletal muscle cells; S6K1, ribosomal protein S6 kinase 1.

Elevated free fatty acid (FFA) levels can cause hyperinsulinemia and insulin resistance in the adipose tissue, increase gluconeogenesis in the liver and suppress glucose utilization in skeletal muscle, accentuating risk of metabolic syndrome [85,86,87,88]. In obese rats fed with HFD, oral treatment with the galectin-1 inhibitor thiodigalactoside (TDG) prevented body weight gain, decreased body fat, glucose, and triglyceride levels. At the protein level, TDG treatment reduced lipogenic and adipogenic markers in white adipose tissue (WAT) and liver when compared to the lean rats and the obese controls groups. Conversely, TDG treatment increased thermogenesis in brown adipose tissue (BAT) activating *Ucp-1* and *Pgc1-α*, both classic markers of thermogenesis [70]. Baek et al. reported that a full knockout of galectin-1 in WAT, BAT and liver tissues of obese mice increased mRNA levels of *Pgc1*-α, *Prdm16* and *Cidea*, in both WAT and BAT [23]. Galectin-1 has also been proposed to regulate adipogenesis and adipose inflammation by binding to CD146 [82]. Thus, it seems that galectin-1 may modulate energy homeostasis in different types of cells. The same study proposed a possible mechanism that galectin-1 modulates the metabolic profile in obese mice. Galectin-1 has affinity to *Pparg*, a regulatory marker for glucose and lipid homeostasis, as well as metabolic inflammation. The authors showed that silencing of galectin-1 reduced *Pparg* whereas overexpression of galectin-1 increased *Pparg* protein expression levels suggesting that galectin-1 promotes fat deposition by activation of *Pparg* [23]. In contrast, treatment in obese mice with rosiglitazone, a *Pparg* agonist, reduced the circulating levels of galectin-1 but had no effect on mRNA levels of galectin-1 in the subcutaneous and visceral adipose tissue. However, a two-week treatment with rosiglitazone in obese healthy participants was enough to up-regulate galectin-1 protein levels [72]. Thus, data on the interaction between galectin-1 and *Pparg* in mice and humans are inconsistent. Together, these observations open the possibility to consider galectin-1 in a clinical perspective since *Pparg* is a key target to improve insulin sensitivity [89]. It will be interesting to explore the effects on galectin-1 levels of other pharmacological agents with known positive metabolic effects in adipose tissue, e.g., metformin.

Studies have indicated an association between galectin-1 and leptin [22]. Leptin is produced and released from adipose tissue and acts on neurons in the hypothalamus to regulate satiety. Deficiency of leptin in human and animal models has been shown to cause hyperphagia [68,90,91,92]. Like galectin-1, leptin is closely associated with obesity and these proteins are also associated with each other. Furthermore, several reports suggest that this association may actually indicate a functional relationship between the two proteins. Galectin-1 knockout in lean and obese mice results in lower serum levels of leptin but no change in body weight [22], and inhibition of galectin-1 by oral treatment with TDG, an inhibitor for several proteins in the galectin family, also reduced plasma levels of leptin [70]. Further, a full knockout of galectin-1 in WAT, BAT and liver tissue of obese mice had strong metabolic effects with reduction in body weight, but similar effects were not observed in lean mice where no change in food intake occurred [23]. These observations could indicate that galectin-1 interacts with leptin signalling, for instance, in the central nervous system, to modulate food intake and satiety. However, studies have yet to report any effects of galectin-1 on leptin signalling in the specific pathways. Further studies are necessary to clarify a relationship between galectin-1 and leptin.

In agreement with the results above, a recent study showed that galectin-1 mRNA levels were abundant in metabolic tissues such as muscle, BAT and WAT [23]. Obese mice had high protein expression levels of galectin-1 in these organs when compared to lean mice. Furthermore, galectin-1 expression increased during differentiation of 3T3L1 adipocytes (a cell line from mouse embryos), while galectin-1 knockout using small interfering RNA (siRNA) in the cells stopped the differentiation to mature adipocytes [23]. Mukherjee and colleagues noted that knockout of galectin-1 in a 3T3-L1 cell line was enough to suppress mRNA levels of lipogenic and adipogenic markers *Pparg*, *Cebpα*, *Fasn* and *Acc* following reduction in the triglyceride content. Similar effects on mRNA levels were found when TDG treatment was administered in mature 3T3L1 adipocytes [70]. The adipocytes are also adaptable and change structure during weight gain when more triglycerides are stored. Several studies have shown that galectin-1 is highly expressed in adipocytes from both humans and animals [15,22,23,24,25,69,70,93]. A study in mice that assessed the regulatory function of galectins noted an increase of galectin-1 mRNA and protein levels specifically in subcutaneous adipocytes but not in visceral adipocytes in obese mice compared to lean control mice [22]. Furthermore, no difference in galectin-1 level was found in the stroma vascular fraction between the groups [22].

## 3. Galectin-1 in Insulin Resistance and Type 2 Diabetes

The scientific evidence for a possible role of galectin-1 as a mediator of pathophysiological mechanisms behind insulin resistance has also increased in recent years, as several clinical studies have presented similar results in this area. One of the first to indicate a role for galectin-1 in insulin resistance was a study of 10 obese but otherwise healthy insulin-resistant men treated with the insulin sensitizing PPAR-γ agonist rosiglitazone (4 mg bid) for 14 days [72]. In an unconditional proteomics analysis of the subcutaneous adipose tissue, galectin-1 levels were doubled during treatment. It was speculated that the insulin sensitizing effect of rosiglitazone could be mediated through a remodulation of the adipose tissue, with a concurrent increased lipid storage, and that galectin-1 is part of this process [72]. To further corroborate this observation, galectin-1 has been associated with serum insulin levels independently of BMI in two large cohort studies [47,64]. Galectin-1 has anti-inflammatory properties [79,83,98] and has also been associated with several inflammatory markers increased in human insulin resistance, including IL-6, TNF-α and CRP [47,64].

Several animal models also demonstrate a role of galectin-1 in insulin resistance. Studies intervening with galectin-1 consistently affect weight loss, with improved metabolic control in obese animals as a consequence. These studies have mainly emphasized the adipose tissue and its role in the effects of galectin-1 on metabolic outcome. Little is therefore known about the potential influence of galectin-1 on metabolic regulation in other metabolically active organs including muscle, liver, kidney, brain and gut. Galectin-1 inhibition through lactulose treatment in Sprague Dawley rats on a HFD improved metabolic control by reducing FFA and triglyceride levels alongside a reduced body weight [24]. Oral administration of lactulose also suppressed fasting insulin levels while increasing the adipose tissue protein expression of the beta-oxidation related protein carnitin-palmitoyl-transferas-1A (CPT1), and the lipolysis regulator hormone sensitive lipase (HSL). These observations indicate a direct metabolic role of adipose tissue galectin-1 in relation to insulin resistance [24]. Adding to these experiments, in Sprague Dawley male rats fed a HFD diet, treatment with another oral galectin-1 inhibitor, lactobionic acid, normalized glucose levels during an intraperitoneal glucose tolerance test, while lowering fasting leptin and triglyceride levels and increasing the fasting glycerol levels, indicating an increase in basal lipolysis [25].

Blocking galectin-1 with TDG also produced similar results. These animals presented with lower blood glucose levels as well as suppressed plasma triglycerides and insulin [70]. These reports indicate several different mechanisms through which galectin-1 could be involved in processes underlying an insulin resistant state. Studies on galectin-1 knockout mice (*Lgals1^−/−^)* indicate interactions between galectin-1 and metabolic inflammation, as well as pancreas function. *Lgals1^−/−^* HFD-fed male mice presented significantly lower levels of fasting glucose, as well as WAT gene expression of pro-inflammatory cytokines including TNF-α and the macrophage marker F4/80, compared to wild type mice [23]. Further, LPS treatment reduced galectin-1 plasma levels in another mouse model [73]. Thus, more studies are required to fully understand the modulatory role of galectin-1 on inflammation and the effect of inflammatory activation on galectin-1. Sundblad et al. studied female *Lgals1^−/−^* mice and could show higher fasting glucose, increased pancreas mass and higher pancreatic insulin content, but reduced serum insulin levels in mice at 2 months of age. However, at this age there was no alteration in glucose or insulin tolerance. Finally, insulin secretion from pancreatic islets in *Lgals1^−/−^* mice increased synergistically compared to wild-type mice after stimulation with recombinant galectin-1 and glucose [80].

While the largest body of evidence for a role of galectin-1 in insulin resistance is currently focused on the adipose tissue, galectin-1 is altered in many different cells in the insulin resistant state. Several studies demonstrate a significant role of galectin-1 in adipocyte metabolism, but there are also studies in muscle, kidney, and inflammatory cells [71,77,95,96,99]. Glucose has been demonstrated to stimulate galectin-1 secretion in several cell types. Rat L6 (skeletal muscle cells) galectin-1 expression is altered depending on media glucose concentrations [71], human podocytes (kidney cells) cultured in the presence of high glucose also increase galectin-1 expression on both gene and protein level [96]. Furthermore, galectin-1 increases in murine proximal tubular cells (MPT, another kidney cell type) after treatment with insulin or when cultured in high glucose concentrations [77], and intracellular galectin-1 increases in renal epithelial cells cultured in high glucose conditions [95].

Other mechanisms linking galectin-1 and adipocytes are seen in 3T3-L1 cultured mice preadipocytes. Here, intracellular galectin-1 levels are increased after treatment with a differentiation cocktail of rosiglitazone, insulin and 3-isobutyl-1-methylxanthine [100]. Treatment with the lipolysis stimulator guggulsterone also increased galectin-1 on protein and gene level in 3T3-L1 cells [100]. Taken together, these studies indicate that galectin-1 expression increases after different treatments in preadipocytes. However, which cells make the largest contribution to the circulating levels of galectin-1 is currently unknown as these observations are from cellular experiments and not directly comparable. Consideration should also be given to a paradoxical discrepancy between an apparent stimulating effect of glucose on galectin-1 production in several cell lines, and a negative association between circulating galectin-1 and fasting glucose in several studies [64,69]. The strong association between galectin-1 and body mass could indicate that elevated levels of galectin-1 reported in obesity are largely explained by cells in the adipose tissue (Figure 1A). However, it is also possible that other tissues or cell types make a significant contribution to the circulating effects, especially in lean individuals with less adipose tissue, or with high levels of stimulating factors such as ongoing inflammation or hyperglycaemia (Figure 1B).

On the protein level, galectin-1 has been demonstrated to interact with key metabolic signalling pathways in several different tissues and through several different proteins downstream of the insulin receptor. Differences in study design may explain the large variety in different signalling pathways proposed for galectin-1 in insulin resistance and highlight the need for additional mechanistic studies elucidating which of these interactions are of highest clinical relevance. Isolated human adipocytes preincubated with recombinant galectin-1 in vitro presented a reduced glucose uptake, independent of insulin dose, but altered *GLUT4* expression [15]. Inhibition of galectin-1 with 30 μM of the inhibitor OTX-008 in MPT cells decreased insulin-induced Akt-phosphorylation [77], and galectin-1 also activates p38 and extracellular signal-regulated kinase (ERK) in human renal epithelial cells [95]. In experiments on embryonic stem cells in mice, galectin-1 stimulated GLUT1-mediated glucose uptake in a dose-dependent manner. The galectin-1 effect on glucose uptake was dependent on PI3K, AKT, mTOR and ERK1/2 [94]. Galectin-1 expression has also been linked to the ribosomal protein S6 kinase 1, downstream mTORC1 in liver and in adipose tissue in mice [84].

**Figure 2:**
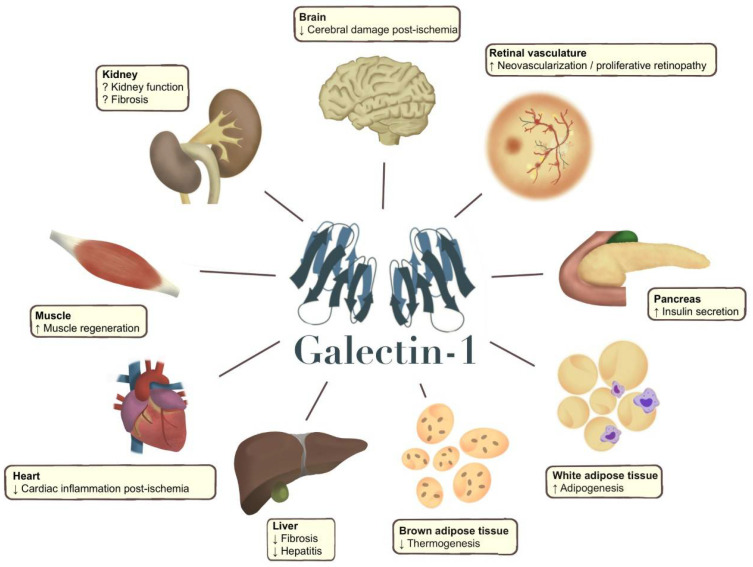
Galectin-1 effects in different organs (arrow up or down) reported in pre-clinical studies (animal models and human cells), which should be confirmed in human tissues and clinical studies. Galectin-1 has been shown to improve tissue recovery after a cerebral stroke [101,102]. Galectin-1 also increases neovascularization and unwanted vascular proliferation in diabetic retinopathy [42,103,104]. In the pancreas, galectin-1 has been shown to stimulate insulin secretion in female mice, and circulating galectin-1 is also positively associated with serum insulin in studies in humans [47,64,80]. Studies have linked galectin-1 to adipogenesis, and it has been proposed that galectin-1 could directly affect *pparg* expression [23,72]. Galectin-1 has also been shown to reduce thermogenesis in mice [23,82]. In the liver, inhibition of galectin-1 results in increased fibrosis and increased severity of hepatitis [105], and galectin-1 reduces inflammation in the heart after ischaemia [106,107]. Galectin-1 also appears to facilitate skeletal muscle regeneration in muscle degenerative conditions, as well as in muscle damage [108]. In the kidney, results have been conflicting, as a Mendelian randomization study in humans and an intervention study in mice have demonstrated kidney protective effects of galectin-1 [43,47]. Conversely, circulating galectin-1 has been associated with a lower kidney function in cross-sectional and longitudinal studies [47,48], and in vitro studies have demonstrated contradicting associations with different fibrotic markers [95,96,109].

Several experimental studies emerging in recent years relate galectin-1 to the pathophysiology behind T2D (Figure 2). The first study identifying altered levels of galectin-1 in T2D used proteomics, revealing elevated circulating galectin-1 levels in T2D. Liu et al. set out to characterize the proteome in plasma from 17 individuals with T2D and compared it to 15 individuals without T2D. Circulating galectin-1 was 4.8-fold higher in participants from the T2D group. Galectin-1 expression experiments in cell lines found that glucose stimulated galectin-1 in L6 skeletal muscle cells in vitro [71]. Based on these observations the authors proposed that galectin-1 is an important regulator of the pathophysiology of T2D and a novel plasma marker of the disease.

In another unconditional proteomics study, galectin-1 was identified as a protein elevated in the subcutaneous interstitial fluid of individuals with T2D. In an effort to characterize the proteome in human subcutaneous interstitial fluid in T2D, Fryk et al. applied monitoring by microdialysis in situ followed by tandem mass spectrometry. The study recruited seven recently diagnosed male T2D patients with heredity for T2D and eight healthy control subjects without diabetes heredity. In silico pathway analysis of the differently expressed proteins in the secretome indicated a possible function in T2D patients related to carbohydrate metabolism, molecular transport, lipid metabolism, and small molecule biochemistry; galectin-1 was one of 30 up-regulated proteins in participants with T2D. The higher galectin-1 levels in subcutaneous dialysates from participants with T2D were confirmed, and *LGALS1* expression was also higher in isolated subcutaneous adipocytes. Furthermore, a strong correlation between dialysate galectin-1 and adipocyte *LGALS1* expression of galectin-1 was observed. This study did not observe any difference in circulating levels of galectin-1 between the groups but did report positive correlations between serum galectin-1 and the insulin resistance related variables fat cell size and waist-hip ratio [15]. The authors concluded that galectin-1 secreted from the subcutaneous adipose tissue may be involved in the development of T2D.

The association between galectin-1 and T2D has also been examined in two population-based studies. The first study measured circulating galectin-1 in 989 individuals in Gothenburg, Sweden at the age of 40–65 years and investigated the association between serum galectin-1 and T2D independently of BMI, excluding individuals with a medical history of cancer or autoimmune diabetes. Using linear regression models, the authors found that serum galectin-1 was inversely associated with T2D in a model adjusted for age, sex, and BMI [64]. The cross-sectional design prevents any conclusions on the causal relationship between galectin-1 and T2D, and it is not known if a lower galectin-1 found in T2D was a contributing cause, or a consequence, of T2D. It would therefore be of interest to measure galectin-1 in prospective cohort studies to monitor the galectin-1 trajectory from normal to prediabetes and newly diagnosed T2D. Simple correlations between serum galectin-1 and HbA1c were again statistically significant, but in BMI-adjusted linear regression models serum galectin-1 was inversely associated with fasting glucose. These observations could indicate a confounding effect of BMI in the relation between galectin-1 and variables of glucose homeostasis [64]. The second study examined the predictive value of galectin-1 on incident T2D using a middle-aged population-based cohort in southern Sweden. Four thousand twenty-two individuals (58.6% women, mean age 57.6 years) were followed over an average of 18.4 years and T2D status was ascertained through registries. Cox regression was used adjusting for established risk factors and galectin-1 was associated with an increased risk of T2D (per SD increase, HR 1.12, 95% CI 1.02–1.24). Interestingly, participants in the lowest quartile of galectin-1 levels at baseline more often reported a heredity for T2D than participants with higher galectin-1. A genome-wide association study (GWAS) on galectin-1 was also performed to identify single nucleotide polymorphisms (SNPs) predicting circulating galectin-1 levels. The SNPs were used in a two-sample Mendelian randomization (MR) analysis to explore a direct causal association between galectin-1 and T2D. One genome-wide significant locus in the galectin-1 gene region was identified (sentinel SNP rs7285699, *p* = 2.4 × 10^−11^). However, the two-sample MR analysis did not ascertain any causal effect of galectin-1 on T2D (*p* = 0.19).

Galectin-1 has also been associated with gestational diabetes, and a proteomic study identified higher levels of galectin-1 protein content in the placental tissue of women with gestational diabetes (GD) [110]. In a separate study on women with GD, the authors demonstrate that circulating galectin-1 increases during normal pregnancy, but that this increase is not seen in women who develop GD [111]. Furthermore, galectin-1 secretion from blood cord cells was increased in the presence of glucose [111]. Taken together, reports of high circulating galectin-1 as a predictor of incident T2D, and lower levels of galectin-1 in manifest T2D could indicate that galectin-1 levels fall during disease development. This has also been seen in a study of patients with ulcerative colitis and the metabolic syndrome. The study found that participants with the most severe degree of metabolic syndrome had the lowest circulating galectin-1 levels, which was also associated with a more severe inflammatory condition [81].

## 4. Galectin-1 and Diabetic Complications

Galectin-1 has also been related to organs affected by T2D. Studies link galectin-1 to the pathophysiological processes in liver fibrosis, hepatocellular cancer, cardiovascular disease and cerebral stroke [101,105,106,112] (Figure 2). However, the most significant role of galectin-1 appears to be related to microvascular complications in the kidneys and eyes. Several studies have demonstrated a role of galectin-1 in the development of kidney disease, specifically linked to diabetes [47,95,96], but also in other contexts [43,48]. While the body of evidence aggregates around a specific role in the pathophysiology of the condition, it is still unclear whether galectin-1 contributes to the progression of the disease or if it presents kidney protective effects.

High plasma galectin-1 level has been reported to be a significant predictor of renal function decline, independently of diabetes and other risk factors, in a longitudinal study of 798 individuals who underwent elective coronary angiography or percutaneous coronary intervention. Participants were stratified by galectin-1 levels, and the authors found that high galectin-1 levels predicted the longitudinal decline in kidney function [48].

A smaller study including eight individuals with diabetic nephropathy and three healthy controls suggested increased levels of galectin-1 in the renal tissue of individuals with diabetic nephropathy [96]. In a proteomics study, galectin-1 was also higher in the kidneys of mice with surgically induced chronic kidney disease [113], and a study on kidney fibrosis in mice reports that galectin-1 levels are significantly higher in the kidneys of animals with both type 1 and T2D. Together, these studies demonstrate increased levels of galectin-1 during the progression of kidney disease, but do not provide insight as to the specific role of galectin-1 in this process. In a study on mice, recombinant galectin-1 administered prior to renal ischaemia-reperfusion injury demonstrated kidney protective effects, possibly through anti-inflammatory mechanisms [43].

In a large cohort-study from southern Sweden, a remarkably strong inverse association was shown between galectin-1 and eGFR, pointing towards an important function for galectin-1 in kidney physiology. However, galectin-1 was not associated with incident CKD in fully adjusted regression models, possibly suggesting that other known risk factors could have a confounding influence on the statistical outcome. Furthermore, a two-sample MR analyses did not ascertain any causal effect of galectin-1 on CKD [47]. However, in a separate MR analysis on 9367 T2D patients from the All New Diabetics In Scania (ANDIS) cohort, analyses were stratified according to four recently proposed subtypes of diabetes [114]. Here, genetically elevated galectin-1 was associated with higher eGFR (*p* = 5.7 × 10^−3^) specifically in individuals with severely insulin-resistant T2D, a group previously known to have an elevated risk of diabetic nephropathy [47]. The authors conclude that galectin-1 is linked to lower kidney function in cross-sectional analyses but that MR analyses suggest a protective effect on kidney function in subjects at high risk of diabetic nephropathy [47].

Several cellular mechanisms have been proposed to explain the potential kidney related effects of galectin-1. In human podocytes, galectin-1 levels increased when cultured under high glucose conditions, while the cellular integrity marker podocin declined. Suppression of galectin-1 levels using siRNA normalized podocin levels in the cells, suggesting a harmful role of galectin-1 for human podocytes in hyperglycaemic conditions. Furthermore, the expression of the fibrosis marker fibronectin increased in murine proximal tubular cells cultured in high glucose conditions but was ameliorated when treated with the galectin-1 inhibitor OTX008, indicating a role of galectin-1 in diabetes induced fibrosis [77]. Galectin-1 expression is also increased in renal epithelial cells cultured during high glucose conditions and further increased by the coincubation with TGF-β1, a fibrotic marker. Conversely, the overexpression of galectin-1 reduced type I collagen expression induced by TGF-β1 stimulation in both normoglycaemic and hyperglycaemic conditions, suggesting a protective effect of galectin-1 against renal fibrosis [95]. Additionally, transient receptor potential vanilloid type 5 (TRPV5) is responsible for Ca^2+^ reabsorption in the distal tubuli, but expression declines during diabetic kidney disease. This may be explained by an increased endocytosis of the protein. Recently, Lee et al. showed that galectin-1 could stabilize TRPV5 on the cell membrane and inhibit harmful endocytosis, thus proposing another kidney protective mechanism in diabetic kidney disease [109]. Taken together, it is becoming clear that high galectin-1 levels observed in the kidney and reflected in the circulation may influence the change in kidney function over time. However, the clinical relevance of the proposed mechanisms is not yet fully understood, and may differ between individuals, even within the large population of individuals with T2D.

There have been several reports on direct and indirect interactions of galectin-1 in VEGF-signalling which make galectin-1 an interesting protein to study in the pathophysiology behind diabetic retinopathy [115,116,117,118]. Galectin-1 levels have also been reported to increase in the vitreous fluid, alongside the progression of diabetic retinopathy in humans. While increased levels are found in diabetic macular oedema, they are not seen in non-diabetic retinopathies such as branch vein retinal occlusion, or central vein retinal occlusion. Through careful in vitro experiments, Kanda et al. propose that galectin-1 is regulated through cellular crosstalk between Müller cells and macrophages through elevated levels of advanced glycation end products (AGEs) and IL-1β [104]. These results have also been corroborated in another study of 20 proliferative diabetic retinopathy (PDR) cases, where plasma galectin-1 was increased, and correlated positively with circulating levels of AGEs, and IL-1β levels [119]. Another study on 13 individuals with PDR confirms elevated levels of galectin-1 in the vitreous fluid. In line with others, the authors examine the role of Müller cells, a glial cell type of the retina, in the elevated levels and propose hypoxia and oxidative stress as promoting factors of this increase [103].

In a study of oxygen-induced retinopathy of mice, galectin-1 levels were increased, and immunostaining indicated increased galectin-1 expression close to retinal neovessels. Furthermore, intravitreal injection of the galectin-1 inhibitor OTX008 significantly reduced the number of preretinal neovascular cells and the retinal neovascularization area, which were otherwise increased in the oxygen-induced retinopathy model [120]. Another study of oxygen-induced retinopathy in mice examined the effect of galectin-1 silencing through intravitreal adenoviral interference injection and discovered that while retinal neovascularization was reduced, there was no negative effect on normal vessel growth or vessel perfusion [42]. Another study on mice observed that elevated levels of galectin-1 persisted after the resolution of vascular alterations. The authors therefore suggest that galectin-1 may also be involved in processes other than neovascularization itself [118]. In line with this report, diabetes-induced retinal expression of galectin-1 can also be suppressed via treatment with anti-inflammatory glucocorticoids in mice, through the modulation of AKT, ERK 1/2 and AP-1 phosphorylation [99].

A study on patients with diabetic retinopathy tested the interaction between galectin-1 and the anti-VEGF treatment Aflibercept. Monthly intravitreal injections with the fusion protein Aflibercept, a protein with similar binding affinities for VEGF receptors 1 and 2, increased the circulating galectin-1 levels over time. The study also demonstrates a direct binding interaction between galectin-1 and Aflibercept, providing insight into a molecular signalling pathway for galectin-1 in diabetic retinopathy [78]. Another study examined the effect of Bevacizumab injection, a monoclonal VEGF-A antibody, on galectin-1 in patients with PDR. The authors reported increased levels of galectin-1 in PDR compared to controls without diabetes but did not find any changes from the Bevacizumab injection. However, the study also reported that the galectin-1 induced VEGFR2 phosphorylation was supressed by co-incubation with Aflibercept in cultured human retinal microvascular endothelial cells. These observations indicate that while galectin-1 can interact with VEGF receptor signalling, the direct interaction with VEGF-A may be limited [117]. Taken together, there are substantial data pointing to a role of galectin-1 in diabetic retinopathy, with promising experimental studies indicating a possible therapeutic potential beside currently available treatment alternatives.

## 5. Summary and Future Prospects

Taken together, studies on human and animal models demonstrate the close association between galectin-1 and pathophysiological processes related to obesity, insulin resistance, and T2D. While observational studies in humans largely align with interventional studies in animal models, it is now important to further validate the potential of intervening with galectin-1 activity as a therapeutic strategy in obesity and T2D in humans. Safety and efficacy studies of galectin-1 blockers to promote weight loss in obese individuals as indicated by several pre-clinical studies could be feasible in the foreseeable future. Of note, there is a discrepancy between different organs regarding how this modulation of galectin-1 should be performed, as it appears that lower levels may be beneficial in weight loss and the treatment of retinopathy, while higher galectin-1 levels may be beneficial for the kidneys and for stimulation of insulin secretion. Thus, it may be necessary to suppress galectin-1 when targeting certain cell types and conditions, while stimulation of the galectin-1 signal may be relevant in other states and cell types. Furthermore, while several studies show an up-regulation of galectin-1 with dietary intake, little is known regarding the impact of physical activity on galectin-1 levels. Other metabolically active peptides, such as irisin, adropin and preptin, have previously been shown to be important peripherally secreted regulators of energy homeostasis, with potential implications for obesity and T2D [121,122]. Mechanistically, several studies associate galectin-1 with important adipokines and transcription factors, including leptin and PPAR-γ [22,23,24,72]. It is thus warranted to continue to evaluate how galectin-1 is connected to secreted proteins and peptides in energy metabolism, and how it translates to metabolic effects *in vivo*. Adipose tissue crosstalk with the brain is important for weight stability, and studies on galectin-1 in the CNS are therefore of interest.

As a role for galectin-1 in insulin resistance becomes more apparent, the distinct role of galectin-1 in the pathophysiological mechanisms in this state appear complex. Disentangling the specific galectin-1 signalling pathways is challenged by the large number of ligands and receptors proposed for galectin-1 [123]. Experimental studies have demonstrated mechanisms in several distinctly different pathways but the translation to clinical samples and validation largely remains. Interesting observations in markers downstream of the insulin- and VEGF-receptors were made [94,116], but how galectin-1 conveys its cellular effects in different organs *in vivo* remains largely unknown and needs to be studied further. High expression of galectin-1 in adipose tissue is well described, but it is also reported that galectin-1 levels are increased in the eye and kidney in T2D [96,104]. The relative contribution of locally produced galectin-1 in various tissues on the circulating levels should thus be characterized *in vivo*. Furthermore, the apparent associations with glucose- and insulin regulation as well as inflammatory markers reported in population-based studies on individuals using BMI-adjusted regression models, as well as in in vitro experiments, highlight a need of additional mechanistic studies to further disentangle these conditions. The metabolic role of galectin-1 in tissues other than the adipose tissue could also provide novel insights into the role of galectin-1 in the development of T2D, as well as in the context of the development of diabetes-related complications (Figure 2). Longitudinal studies on individuals converting from prediabetes to T2D should include repeated measurements of serum galectin-1. Of note, there is a higher prevalence of heredity for type 2 diabetes in individuals with low galectin-1 levels [47].

Galectin-1 has an important role in tissue remodelling and regeneration [1]. The altered regulation of galectin-1 in obesity and metabolic disease could therefore also have an impact on several complications related to T2D. While we have already discussed the potential influence of galectin-1 in diabetic kidney disease and retinopathy, galectin-1 has also been linked to lymphatic remodelling [76], although not in the context of T2D. Studies of galectin-1 have previously indicated altered regulation in both cerebral stroke, myocardial infarction, and congestive heart failure [102,106,107,124]. Galectin-1 also has a role in the regeneration of peripheral nerves [125] and wound healing [126], which could be relevant in diabetic peripheral neuropathy and diabetic foot ulcers. Going forward, advancing the knowledge around galectin-1 in obesity and metabolic disease could further shed light on the pathophysiology of T2D and its complications and help to find out whether galectin-1 has potential as a biomarker in prediabetes and T2D. Knowledge of several established and proposed ligands of galectin-1 enables the future identification of pathway-specific promotors of beneficial galectin-1-regulated effects, while pharmacological inhibitors of galectins are already in early clinical trials [4,6].
